# Selective Packaging in Murine Coronavirus Promotes Virulence by Limiting Type I Interferon Responses

**DOI:** 10.1128/mBio.00272-18

**Published:** 2018-05-01

**Authors:** Jeremiah Athmer, Anthony R. Fehr, Matthew E. Grunewald, Wen Qu, D. Lori Wheeler, Kevin W. Graepel, Rudragouda Channappanavar, Aimee Sekine, Dana Saud Aldabeeb, Michael Gale, Mark R. Denison, Stanley Perlman

**Affiliations:** a Department of Microbiology and Immunology, University of Iowa, Iowa City, Iowa, USA; b Department of Pathology, Microbiology and Immunology, Vanderbilt University Medical Center, Nashville, Tennessee, USA; c Department of Pediatrics, Vanderbilt University Medical Center, Nashville, Tennessee, USA; d Elizabeth B. Lamb Center for Pediatric Research, Vanderbilt University Medical Center, Nashville, Tennessee, USA; e Center for Innate Immunity and Immune Disease, Department of Immunology, University of Washington School of Medicine, Seattle, Washington, USA; f Department of Medicine, King Saud University Medical City, College of Medicine, Riyadh, Saudi Arabia; Icahn School of Medicine at Mount Sinai

**Keywords:** coronavirus, interferon response, murine hepatitis virus, RNA packaging, packaging signal, selective packaging

## Abstract

Selective packaging is a mechanism used by multiple virus families to specifically incorporate genomic RNA (gRNA) into virions and exclude other types of RNA. Lineage A betacoronaviruses incorporate a 95-bp stem-loop structure, the packaging signal (PS), into the nsp15 locus of ORF1b that is both necessary and sufficient for the packaging of RNAs. However, unlike other viral PSs, where mutations generally resulted in viral replication defects, mutation of the coronavirus (CoV) PS results in large increases in subgenomic RNA packaging with minimal effects on gRNA packaging *in vitro* and on viral titers. Here, we show that selective packaging is also required for viral evasion of the innate immune response and optimal pathogenicity. We engineered two distinct PS mutants in two different strains of murine hepatitis virus (MHV) that packaged increased levels of subgenomic RNAs, negative-sense genomic RNA, and even cellular RNAs. All PS mutant viruses replicated normally *in vitro* but caused dramatically reduced lethality and weight loss *in vivo*. PS mutant virus infection of bone marrow-derived macrophages resulted in increased interferon (IFN) production, indicating that the innate immune system limited the replication and/or pathogenesis of PS mutant viruses *in vivo*. PS mutant viruses remained attenuated in MAVS^−/−^ and Toll-like receptor 7-knockout (TLR7^−/−^) mice, two well-known RNA sensors for CoVs, but virulence was restored in interferon alpha/beta receptor-knockout (IFNAR^−/−^) mice or in MAVS^−/−^ mice treated with IFNAR-blocking antibodies. Together, these data indicate that coronaviruses promote virulence by utilizing selective packaging to avoid innate immune detection.

## IMPORTANCE

Coronaviruses (CoVs) produce many types of RNA molecules during their replication cycle, including both positive- and negative-sense genomic and subgenomic RNAs. Despite this, coronaviruses selectively package only positive-sense genomic RNA into their virions. Why CoVs selectively package their genomic RNA is not clear, as disruption of the packaging signal in MHV, which leads to loss of selective packaging, does not affect genomic RNA packaging or virus replication in cultured cells. This contrasts with other viruses, where disruption of selective packaging generally leads to altered replication. Here, we demonstrate that in the absence of selective packaging, the virulence of MHV was significantly reduced. Importantly, virulence was restored in the absence of interferon signaling, indicating that selective packaging is a mechanism used by CoVs to escape innate immune detection.

## INTRODUCTION

Coronaviruses (CoVs), like all positive-single-stranded RNA (+ssRNA) viruses, replicate in the cytoplasm of the host cell on restructured membranes (1, 2). In the case of coronaviruses, replication/transcription complexes (RTCs) assemble on endoplasmic reticulum (ER) membranes. The RTC, a multiprotein complex, replicates genomic RNA (gRNA) and transcribes subgenomic RNA (sgRNA) during viral infection. During replication, different viral RNAs are replicated, transcribed, translated, or assembled into virions. In RNA virus infections, replication and assembly are often coordinated by *cis*-acting RNA elements, which may undergo intramolecular RNA interactions to form secondary structures important for recognition. Concordantly, during coronavirus infection, gRNA and sgRNAs must be distinguishable for the purposes of replication, transcription, translation, and assembly.

CoVs are very adept at packaging only gRNA into virions, while sgRNAs are almost completely excluded from virions, despite having identical 5′ and 3′ ends as the gRNA. In lineage A betacoronaviruses (β-CoVs), such as murine hepatitis virus (MHV), the selective packaging of gRNA is dependent on the packaging signal (PS) and its interaction with structural proteins. Early studies of the MHV PS identified a 190-bp region within the nsp15 locus as the PS (3). In both infection and virus-like particle systems, the 190-bp PS was sufficient for packaging of nonviral RNA (4). Selective packaging is intimately tied to interactions with the structural proteins N and M. The selective packaging of nonviral RNA was correlated with M protein binding (5). Furthermore, N protein binds to RNA indiscriminately and was not required for packaging of nonviral RNA. However, when the N protein C-terminal domain (CTD) from MHV was swapped with sudden acute respiratory syndrome-associated CoV (SARS-CoV), significant increases in packaged sgRNAs were observed (6). These two studies propose two different models for selective packaging with M, or alternatively N, protein playing the dominant role in conferring packaging selectivity.

**FIG 1:**
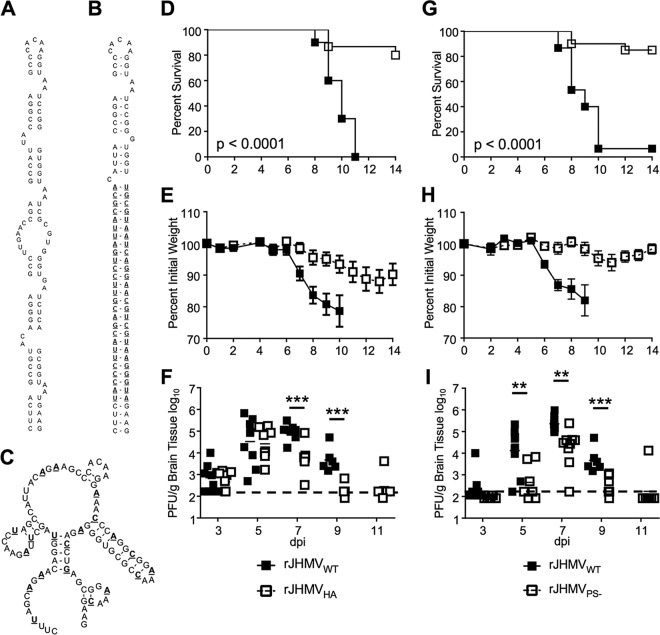
rJHMV packaging mutants are attenuated *in vivo*. (A) The RNA secondary structure of the MHV packaging signal (7). (B) Mfold predicted RNA secondary structure of rA59_Nsp15-HA_. HA epitope coding sequence and complement are boldfaced and underlined. (C) Mfold predicted RNA secondary structure of MHV PS^−^. Synonymous mutations are boldfaced and underlined. (D to F) Five- to 8-week-old male B6 mice were infected with 3 × 10^4^ PFU of rJHMV_Nsp15-HA_ or rJHMV_WT_ by intranasal inoculation. Infected mice were monitored for survival (D) and weight loss (E) for 14 dpi (rJHMV_WT_, *n* = 10; rJHMV_Nsp15-HA_, *n* = 15). (F) Infected brains were harvested from mice at the indicated day postinfection. Brains were homogenized in PBS, and titers of infectious virus were determined by plaque assay on HeLa-MHVR cells. (G to I) Five- to 8-week-old male B6 mice were infected with 3 × 10^4^ PFU of either rJHMV_PS−_ or rJHMV_WT_ by intranasal inoculation. Infected mice were monitored for survival (G) and weight loss (H) for 14 dpi (rJHMV_WT_, *n* = 15; rJHMV_PS−_, *n* = 20). (I) Infected brains were harvested from mice at the indicated day postinfection. Brains were homogenized in PBS, and titers of infectious virus were determined by plaque assay on HeLa-MHVR cells. The dashed line in panels F and I represents the limit of detection for the plaque assay. *, *P* < 0.05; **, *P* < 0.01; ***, *P* < 0.001 by Mann-Whitney test.

A subsequent report on the PS identified a 95-bp stem-loop structure within the previously described 190-bp region (7). Structural study of the PS from MHV, human coronavirus HKU1 (HCoV-HKU1), bovine CoV, and HCoV-OC43 revealed several conserved elements within the 95-bp stem-loop structure, including 2-nucleotide (nt) bulges, AGC/GUAAU motifs, and a pentaloop (Fig. 1A) (7). More recent studies utilizing 20 synonymous mutations predicted to abolish the PS stem-loop structure demonstrated that the secondary structure of the PS was necessary for selective packaging during MHV infection (8). In this instance, significantly higher levels of sgRNA were packaged into PS mutants than in wild-type MHV. Remarkably, this increase in sgRNA did not accompany equivalent decreases in packaging of gRNA or in viral titers (8). Thus, these PS mutants had no defect in virus production in tissue culture cells compared to wild-type MHV (8). This is in contrast to the role of the alphavirus packaging signal, where PS mutants also lead to increased sgRNA packaging, but at the expense of gRNA packaging and viral fitness in cultured cells (9). Retrovirus packaging signal mutants also lead to greatly decreased genomic RNA packaging with a concomitant increase in cellular mRNA packaging (10).

Since PS mutations in non-CoVs result in large decreases in gRNA incorporation into virions and diminished replication, infection with lineage A betacoronaviruses provides a unique opportunity to study the effect of selective viral packaging *in vivo*. Here, using two different PS mutants, we demonstrate that disrupting the selective packaging of MHV resulted in highly attenuated viruses *in vivo*, and this was true for A59 and JHMV, two different strains of MHV. Furthermore, the PS was important for repressing the type I interferon (IFN-I) response during MHV infection of bone marrow-derived macrophages (BMMs). Finally, IFN-I signaling was essential for the attenuation of selective packaging mutants as the virulence of P/S mutants was restored in interferon alpha/beta receptor-knockout (IFNAR^−/−^) mice or mice treated with IFNAR-blocking antibody. This study establishes the PS as a novel virulence factor for MHV, and likely other coronaviruses.

## RESULTS

### MHV packaging signal mutants are attenuated *in vivo*.

Previously, we inserted the RNA sequence encoding a hemagglutinin (HA) tag within the PS of the nsp15 locus (shown in Fig. 1A and B). This virus, termed rA59_Nsp15-HA_, replicated like wild-type virus but had increased levels of packaged sgRNA, indicating that selective packaging of gRNA was disrupted (11). Based on these observations, we next investigated the effect of selective packaging on virulence. B6 male mice were infected with rA59_Nsp15-HA_ by intracranial injection and monitored for morbidity and mortality for 12 days postinfection (dpi). rA59_Nsp15-HA_-infected mice had decreased weight loss and increased survival compared to wild-type rA59 (rA59_WT_) (see Fig. S1A and B in the supplemental material). To test whether this phenotype was strain specific, we introduced the HA sequence into nsp15 of the rJHMV strain of MHV (rJHMV_Nsp15-HA_), using a previously described full-length pBAC-based reverse genetics system (12). rJHMV_Nsp15-HA_ had growth kinetics *in vitro* equivalent to that of rJHMV_WT_ (Fig. S2A), as previously described for rA59_Nsp15-HA_-infected cells (11). Mice infected with rJHMV_Nsp15-HA_ had substantially decreased weight loss and increased survival (Fig. 1D and E). While these data suggest that selective packaging is important for MHV virulence, we cannot rule out subtle effects of the HA tag on the function of nsp15 endoribonuclease, a known IFN-I antagonist. Further, addition of the HA sequence resulted in a 31-bp double-stranded RNA (dsRNA) stem, which could conceivably induce an innate immune response (13, 14).

10.1128/mBio.00272-18.1 FIG S1  rA59 packaging mutants are attenuated *in vivo*. (A and B) Five- to 8-week-old male B6 mice were infected by intracranial inoculation of 700 PFU of rA59_Nsp15-HA_ or rA59_WT_ and monitored for survival (A) and weight loss (B) for 10 dpi (rA59_WT_, *n* = 10; rA59_Nsp15-HA_, *n* = 10). (C and D) Five- to 8-week-old B6 mice were infected by intracranial inoculation of 700 PFU of rA59_PS−_ or rA59_WT_ and monitored for weight loss (C) and survival (D) for 12 dpi (rA59_WT_, *n* = 10; rA59_PS−_, *n* = 10). Download FIG S1, TIF file, 0.4 MB. Copyright © 2018 Athmer et al. 2018 Athmer et al. This content is distributed under the terms of the Creative Commons Attribution 4.0 International license.

10.1128/mBio.00272-18.2 FIG S2  rJHMV packaging mutants replicate the same as wild-type virus in tissue culture cells. (A and B) 17Cl-1 cells were infected at an MOI of 0.1 PFU/cell with rJHMV_WT_, rJHMV_Nsp15-HA_ (A), or rJHMV_PS−_ (B), and viral progeny were collected at the indicated time points. Input virus was collected following the adsorption step. Virus titers were determined by plaque assay on HeLa-MHVR cells. The average and range for three biological replicates are plotted from a single experiment. Data are representative of two independent experiments. 3.2 and 4.2 represent two separate clones of rJHMV_PS−_. Download FIG S2, TIF file, 0.3 MB. Copyright © 2018 Athmer et al. 2018 Athmer et al. This content is distributed under the terms of the Creative Commons Attribution 4.0 International license.

To separate the roles of nsp15 protein and of the PS in virulence, we constructed a second set of viruses with packaging defects in the context of both rA59 and rJHMV (termed PS^−^), as previously described (8). These mutants have synonymous mutations throughout the PS, which are predicted to alter the stem-loop structure of the PS without changing the amino acid sequence of nsp15 (Fig. 1C). The growth kinetics of two independent rJHMV_PS−_ clones (3.2 and 4.2) was nearly identical to that of rJHMV_WT_ *in vitro* (Fig. S2B). These data are consistent with previous results using rA59_PS−_ and indicate that the PS has few, if any, effects on replication *in vitro* (8). After confirming the fitness of rJHMV_PS−_ *in vitro*, we next assessed the virulence of rJHMV_PS−_ in infected mice. B6 mice were infected intranasally with either rJHMV_WT_ or rJHMV_PS−_ and monitored for morbidity and mortality for 14 dpi. Intranasal infection of rJHMV_WT_ resulted in nearly 100% mortality (Fig. 1G) and substantial weight loss (Fig. 1H). Mice infected with rJHMV_PS−_ had significantly increased survival (Fig. 1G) and reduced weight loss (Fig. 1H). In agreement with results using rJHMV, mice infected with rA59_PS−_ also had decreased weight loss and increased survival compared to mice infected with rA59_WT_ (Fig. S1C and D).

We next measured the viral load of rJHMV_WT_, rJHMV_PS−_, and rJHMV_Nsp15-HA_ in the brains of infected mice. Both rJHMV_WT_ and rJHMV_Nsp15-HA_ invaded the central nervous system (CNS) within 3 dpi and replicated to similar levels up to 5 dpi (Fig. 1F). By 7 dpi, titers in rJHMV_Nsp15-HA_-infected mice were decreased in the CNS compared to those in mice infected with rJHMV_WT_. rJHMV_Nsp15-HA_ was cleared by 9 dpi. rJHMV_PS−_ also invaded the CNS, albeit with a delay, and reached similar peak titers as rJHMV_Nsp15-HA_ but not rJHMV_WT_ (Fig. 1I). rJHMV_PS−_ titers decreased on 9 dpi and were cleared by 11 dpi (Fig. 1I). Thus, both PS mutant viruses did not reach the same peak viral titers as, and were cleared more rapidly than, wild-type JHMV. These data, utilizing two distinct PS mutant viruses, indicate that selective packaging of viral genomic RNA is essential for optimal virulence in multiple models of MHV infection.

### rA59_PS−_ and rA59_Nsp15-HA_ have increased sgRNA and cellular RNA packaged into virions.

**FIG 2:**
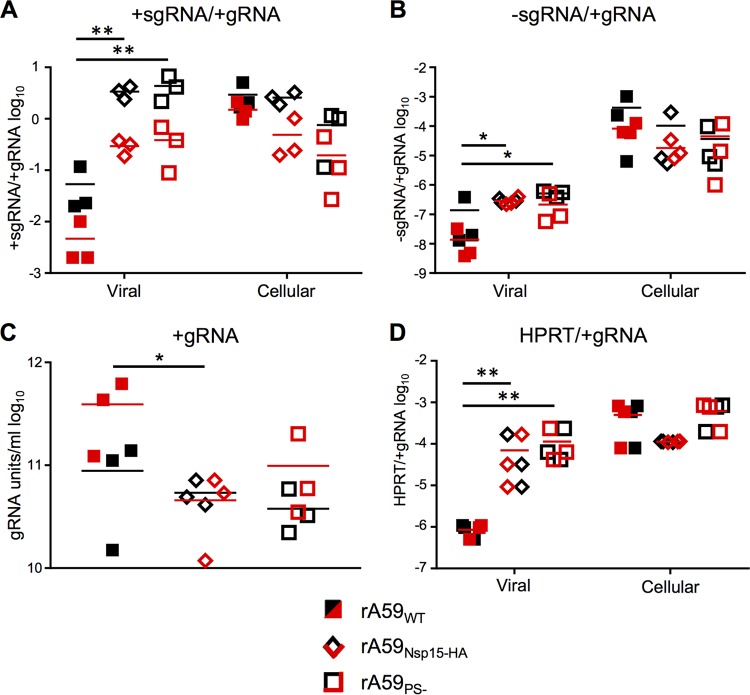
rA59_PS−_ and rA59_Nsp15-HA_ have increased sgRNA and cellular RNA packaged into virions. (A to C) Supernatants from rA59_PS−_-, rA59_Nsp15-HA_-, and rA59_WT_-infected 17Cl-1 cells were collected, and cell debris was removed. Virions were pelleted by ultracentrifugation through a 30% sucrose cushion, and viral RNA was isolated. Intracellular RNA was isolated from the 17Cl-1 monolayers using Trizol. The levels of +sgRNA7, −sgRNA7, and gRNA in concentrated virus and intracellular RNA were measured using RT-qPCR standard curves. The ratios of +sgRNA7 (A) and −sgRNA7 (B) to gRNA were calculated and plotted for viral and intracellular RNA. (C) The number of gRNA units per milliliter was calculated and plotted for each sample. (D) HPRT and gRNA were measured by RT-qPCR. Six biological replicates from two independent experiments are plotted; each experiment is plotted as either red or black. The ratio of HPRT to gRNA was calculated and plotted for viral and intracellular RNA. *, *P* < 0.05; **, *P* < 0.01 by Mann-Whitney test.

We next assessed the degree of the selective packaging defect observed in rA59_PS−_ and rA59_Nsp15-HA_ virions (Fig. 2). To accomplish this goal, the levels of positive- and negative-strand sgRNA7 (+sgRNA7 and –sgRNA7, respectively) and positive-strand gRNA (gRNA) were determined using concentrated virus samples prepared by velocity centrifugation of infected-cell supernatants. In agreement with previous publications (8, 11), both rA59_PS−_ and rA59_Nsp15-HA_ virions had higher levels of packaged +sgRNA7 than gRNA compared to rA59_WT_ virions (Fig. 2A). Furthermore, the ratio of +sgRNA to gRNA in rA59_PS−_ and rA59_Nsp15-HA_ virions was nearly equivalent to the intracellular ratio in rA59_PS−_-infected and rA59_Nsp15-HA_-infected cells. This enrichment of +sgRNA7 to gRNA in rA59_PS−_ and rA59_Nsp15-HA_ virions contrasts with rA59_WT_, where the +sgRNA7-to-gRNA ratio was dramatically decreased compared to the intracellular ratio (Fig. 2A). An increased ratio of −sgRNA7 to gRNA was also observed in rA59_PS−_ and rA59_Nsp15-HA_ compared to rA59_WT_ virions (Fig. 2B) but was not equivalent to the intracellular ratio of these RNAs. Finally, these increases in sgRNA7 incorporation had little effect on the levels of gRNA (Fig. 2C), as neither rA59_PS−_ nor rA59_Nsp15-HA_ virions had more than an ~2-fold decrease in packaged gRNA compared to rA59_WT_ virions.

In retroviruses, the loss of gRNA packaging results in robust packaging of cellular RNA (10). To determine the extent of cellular RNA incorporation by MHV selective packaging mutants, we measured the ratio of hypoxanthine-guanine phosphoribosyltransferase (HPRT) RNA to gRNA in concentrated virus and intracellular RNA samples. We found a significant increase in the HPRT-to-gRNA ratio in both rA59_PS−_ and rA59_Nsp15-HA_ (Fig. 2D) compared to rA59_WT_ virions. However, the ratio of HPRT to gRNA in rA59_PS−_ and rA59_Nsp15-HA_ virions was not equivalent to the intracellular ratio of HPRT to gRNA. These data establish that rA59_PS−_ and rA59_Nsp15-HA_ were similarly defective in selective packaging and suggest that cellular RNA can be packaged into virions at increased levels in selective packaging mutants.

### rA59_PS−_ induces increased levels of IFN-I in BMMs.

**FIG 3:**
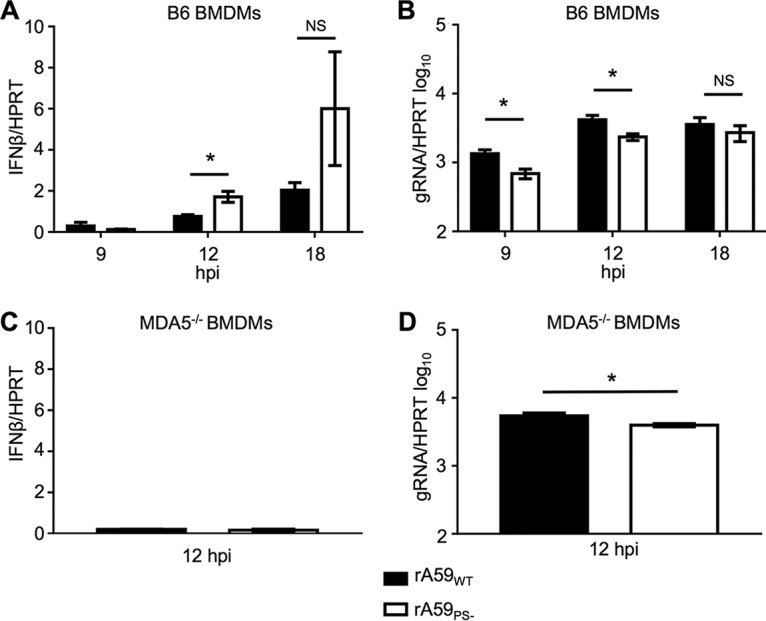
The PS is required to suppress IFN production in bone marrow-derived macrophages (BMDMs). Cultured BMDMs from B6 or MDA5^−/−^ mice were infected at an MOI of 1 PFU/cell with either rA59_WT_ or rA59_PS−_, and RNA was isolated at the indicated time points. The levels of IFN-I (A and C) and gRNA (B and D) were measured by qRT-PCR and normalized to HPRT levels. The average and standard error of the mean for six biological replicates are plotted from two independent experiments.

Because the two selective packaging mutants have similar phenotypes *in vivo* and similar packaging defects, we utilized only rA59_PS−_ and rJHMV_PS−_ to further investigate the mechanism of PS-mediated virulence. The elevated levels of sgRNA, especially –sgRNA, in virions may act as pathogen-associated molecular patterns (PAMPs), which may be sensed during entry, replication, or packaging of PS mutants. To initially test this hypothesis, we utilized bone marrow-derived macrophages (BMMs) because they are easily culturable, are highly susceptible to MHV, and are known to express IFN-I following infection (15). BMMs infected with rA59_PS−_ had an ~2- to 3-fold increase in beta interferon (IFN-β) at 12 h postinfection (hpi) compared to rA59_WT_-infected BMMs (Fig. 3A). The increased IFN-I response was not due to increased replication of rA59_PS−_ as rA59_PS−_ infection resulted in a small (~1.5- to 2-fold) reduction in gRNA (Fig. 3B). In agreement with previous reports, we found that IFN-I production was dependent on MDA5 for both rA59_WT_ and rA59_PS−_ (Fig. 3C). Furthermore, there was still a small difference (~1.5-fold) in replication of rA59_PS−_ in MDA5^−/−^ cells (Fig. 3D). These results indicate that alteration of the packaging signal in MHV can lead to an enhanced innate immune response and perhaps a modest defect in replication in primary cells.

### IFN-I signaling is required for rJHMV_PS−_ attenuation.

**FIG 4:**
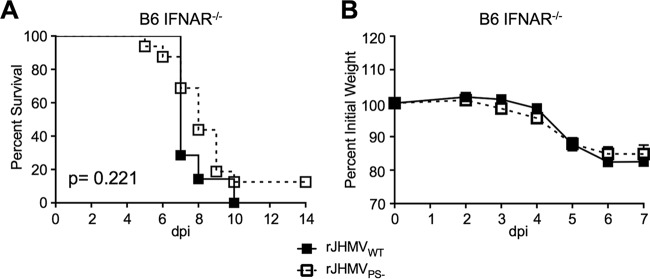
IFN-I signaling is required for rJHMV_PS−_ attenuation. (A and B) Five- to 8-week-old male IFNAR^−/−^ B6 mice were infected with 3 × 10^4^ PFU of rJHMV_PS−_ or rJHMV_WT_ by intranasal inoculation. Infected mice were monitored for survival (A) and weight loss (B) for 14 dpi (rJHMV_WT_, *n* = 7; rJHMV_PS−_, *n* = 14). *, *P* < 0.05 by Mann-Whitney test.

Increased levels of IFN-I in BMMs following rA59_PS−_ infection and the well-established protective role of IFN-I signaling in MHV infection (16) suggested that IFN-I signaling would be important for rA59_PS−_ or rJHMV_PS−_ attenuation. Since rJHMV was more virulent than rA59, rJHMV_PS−_ was used to test this hypothesis. IFNAR^−/−^ mice were infected with rJHMV_PS−_ or rJHMV_WT_ and monitored for morbidity and mortality for 14 dpi. rJHMV_WT_-infected IFNAR^−/−^ mice had substantial weight loss and succumbed to infection earlier than rJHMV_WT_-infected wild-type mice (Fig. 4A and B compared to Fig. 1D and G). IFNAR^−/−^ mice infected with rJHMV_PS−_ at the standard dose of 3 × 10^4^ PFU succumbed to infection in a manner similar to infection with rJHMV_WT_ (Fig. 4A and B). However, at a low dose of virus, 1 × 10^3^ PFU, the virulence of rJHMV_PS−_ was not completely restored (Fig. S3). The increased levels of IFN-β in rA59_PS−_-infected BMMs and rescued virulence of rJHMV_PS−_ in IFNAR^−/−^ mice suggest that IFN-I signaling plays an important role in the attenuation of rJHMV_PS−_. Additionally, other factors, such as delayed neuroinvasion, may also be important for the attenuation of PS^−^ viruses.

10.1128/mBio.00272-18.3 FIG S3  rJHMV_PS−_ virulence is not fully restored in IFNAR^−/−^ mice after infection with a lower dose of virus. (A and B) Five- to 8-week-old male IFNAR^−/−^ B6 mice were infected with 1 × 10^3^ PFU of rJHMV_PS−_ or rJHMV_WT_ by intranasal inoculation. Infected mice were monitored for survival (A) and weight loss (B) for 14 dpi (rJHMV_WT_, *n* = 6; rJHMV_PS−_, *n* = 10). Download FIG S3, TIF file, 0.3 MB. Copyright © 2018 Athmer et al. 2018 Athmer et al. This content is distributed under the terms of the Creative Commons Attribution 4.0 International license.

**FIG 5:**
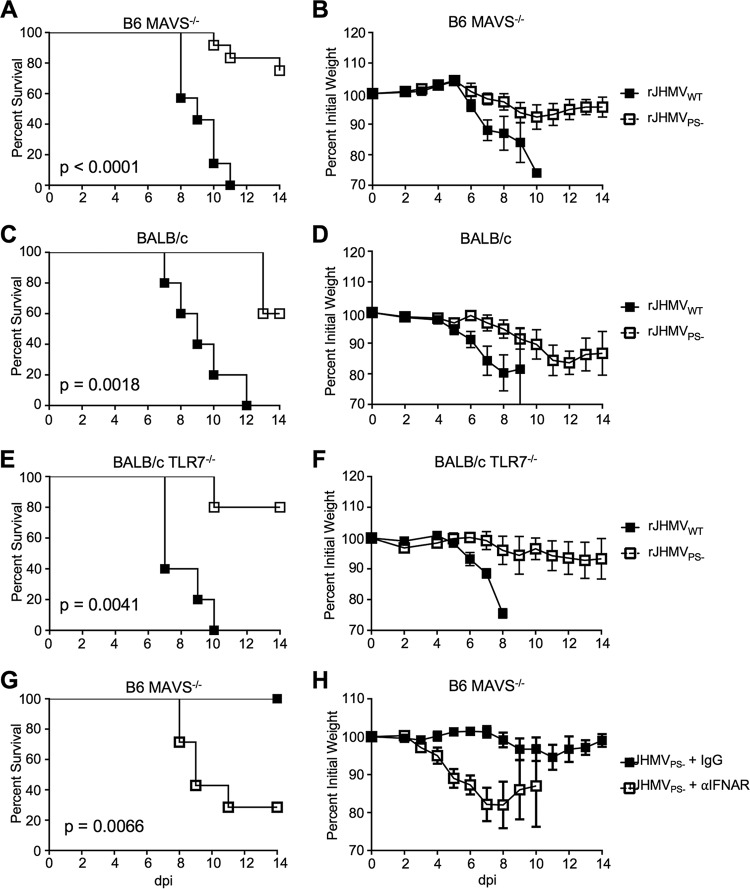
IFN-I signaling during rJHMV_PS−_ is not solely dependent upon either TLR7- or MAVS-mediated signaling. (A and B) Five- to 8-week-old male MAVS^−/−^ mice were infected with 3 × 10^4^ PFU of either rJHMV_PS−_ or rJHMV_WT_ by intranasal injection. Infected mice were monitored for survival (A) and weight loss (B) (rJHMV_WT_, *n* = 7; rJHMV_PS−_, *n* = 12). (C and D) Five- to 8-week-old male BALB/c mice were infected with 1 × 10^4^ PFU of either rJHMV_PS−_ or rJHMV_WT_ by intranasal inoculation. Infected mice were monitored for survival (C) and weight loss (D) (rJHMV_WT_, *n* = 5; rJHMV_PS−_, *n* = 5). (E and F) Five- to 8-week-old male TLR7^−/−^ (BALB/c) mice were infected with 1 × 10^4^ PFU of either rJHMV_PS−_ or rJHMV_WT_ by intranasal inoculation. Infected mice were monitored for survival (E) and weight loss (F) (rJHMV_WT_, *n* = 5; rJHMV_PS−_, *n* = 5). (G and H) Five- to 8-week-old male MAVS^−/−^ (B6) mice were infected with 3 × 10^4^ PFU of rJHMV_PS−_ by intranasal injection. At the time of inoculation and at 3 dpi, 1.5 µg and 0.5 µg of IFNAR-blocking antibody were administered, respectively, by the i.p. route to infected mice, which were monitored for survival (G) and weight loss (H) (rJHMV_WT_, *n* = 7; rJHMV_PS−_, *n* = 7).

Previous work has demonstrated protective roles for MDA5 and Toll-like receptor 7 (TLR7) during coronavirus infection (15, 17). Next, we utilized MAVS^−/−^ mice to determine the role of MDA5-mediated signaling in attenuating rJHMV_PS−_. rJHMV_WT_ caused a lethal infection in MAVS^−/−^ mice, but rJHMV_PS−_ remained attenuated, causing moderate weight loss and no decrease in survival (Fig. 5A and B). MHV induces significant levels of IFN-I in plasmacytoid dendritic cells via TLR7 signaling, contributing to protection (17). To determine the role of TLR7 signaling in protection, we infected TLR7^−/−^ mice with rJHMV_PS−_. Since the TLR7^−/−^ mice that were used were on a BALB/c background, we first confirmed that BALB/c mice were protected from rJHMV_PS−_-mediated lethality. rJHMV_WT_ infection caused complete lethality and substantial weight loss, while rJHMV_PS−_-infected BALB/c mice had a phenotype similar to that observed in rJHMV_PS−_-infected B6 mice, with increased survival and less weight loss (Fig. 5C and D). TLR7^−/−^ mice infected with rJHMV_WT_ succumbed to infection, while we observed no increase in weight loss or lethality after infection with rJHMV_PS−_ (Fig. 5E and F). These results suggest that neither signaling through MAVS nor that through TLR7 is solely responsible for attenuation of packaging signal mutants and suggest that initiation of IFN-I signaling occurs via alternate sensors or is multifactorial.

While neither TLR7 nor MAVS signaling was solely responsible for rJHMV_PS−_ attenuation, it is possible that a protective level of IFN-I was still produced in infected MAVS^−/−^ or TLR7^−/−^ mice. IFNAR^−/−^ mice are incapable of IFN-I signaling and therefore lack expression of interferon-stimulated genes (ISGs). In contrast, MDA5^−/−^ mice infected with MHV had decreased but detectable levels of IFN-I, and the expression of many ISGs was not altered during MHV infection of MDA5^−/−^ mice (18). Next, to determine if IFN-I signaling during infection was responsible for protecting mice or if basal levels of ISGs were playing a significant role, we treated MAVS^−/−^ mice with an IFNAR-blocking antibody (19). In these experiments, IFNAR-blocking antibody was injected intraperitoneally 6 h prior to infection and again 3 days later. Control MAVS^−/−^ mice administered rat IgG remained protected from rJHMV_PS−_; conversely, MAVS^−/−^ mice administered IFNAR-blocking antibody displayed increased weight loss and succumbed to infection (Fig. 5G and H). These data suggest that IFN-I signaling during infection is important for the attenuation of rJHMV_PS−_.

## DISCUSSION

Due to the largely unaltered growth of packaging mutants *in vitro*, the lineage A betacoronaviruses, specifically MHV, provide a unique opportunity to study the effect of viral packaging on virulence. This is not possible in both alphaviruses and retroviruses since packaging mutants have significant decreases in gRNA incorporation into virions and in infectivity (9, 10). Our data identify the lineage A betacoronavirus PS as a novel *cis*-acting virulence factor in MHV.

While the function of the lineage A betacoronavirus packaging signal is well understood *in vitro*, its relevance *in vivo* has not been addressed. The location of the stem-loop structure within the nsp15 locus allows these coronaviruses to selectively package gRNA and not coterminal sgRNA or host RNA. In this work, we quantified the packaging defect in rA59_Nsp15-HA_ and rA59_PS−_. The defects present in these two different PS mutants were strikingly similar, despite rA59_Nsp15-HA_ maintaining the overall structure, the pentaloop, and one of the 2-nucleotide (nt) bulges, elements hypothesized to be important for packaging (Fig. 1A and B). In agreement with previous publications (8), we found a significant increase in sgRNA packaging in both rA59_PS−_ and rA59_Nsp15-HA_ virions. We further divided the sgRNA into +sgRNA and −sgRNA and found increased levels of both in the virions of mutant viruses compared to parental viruses. Furthermore, we found increased levels of host cellular RNA in both packaging mutant virions. Interestingly, while the ratios of +sgRNA7 to gRNA in the virions of both rA59_PS−_ and rA59_Nsp15-HA_ were equivalent to the intracellular ratio, this was not true for ratios of –sgRNA7 or glyceraldehyde-3-phosphate dehydrogenase (GAPDH) to gRNA.

Packaging occurs through an interaction between the PS stem-loop and coronavirus structural proteins. A direct interaction between the M protein and the PS was previously reported and correlated with the packaging of nonviral RNA (20). It can be hypothesized that in the absence of M-PS interactions, packaging of RNA is dependent on N protein. Since N protein binds to RNA indiscriminately (5), levels of packaged RNA would be predicted to closely mimic those present in the intracellular environment. However, the ratio of both –sgRNA7 and GAPDH to gRNA was increased in virions but did not reach intracellular ratios, suggesting that RNA binding by N is not the sole determinant of RNA packaging in rA59_PS−_ and rA59_Nsp15-HA_. RNA localization, secondary structure in other regions of the genome, or other proteins may influence RNA packaging. The relative lack of –sgRNA7 packaging may reflect its localization to membranous structures associated with replication while +sgRNA7 would be exported to the cytosol.

All packaging mutants tested were attenuated in mice. rJHMV_PS−_ and rJHMV_Nsp15-HA_, and rA59_PS−_ and rA59_Nsp15-HA_, were attenuated in the context of lethal encephalitis (Fig. 1) and hepatitis (see Fig. S1 in the supplemental material), respectively. It can be hypothesized that packaged sgRNA and/or host RNAs behave as PAMPs, eliciting an increased IFN-I response in rJHMV_PS−_-infected and rJHMV_Nsp15-HA_-infected cells. Consistent with this hypothesis, IFN-I signaling was essential for this attenuation *in vivo* (Fig. 3). Furthermore, BMMs infected with rJHMV_PS−_ expressed higher levels of IFN-I than rJHMV_WT_ (Fig. 3A). These data indicate that packaging mutants are capable of inducing an increased IFN-I response, which may be protective *in vivo*. We investigated the two best-described sensors for IFN-I signaling during coronavirus infection, MDA5 and TLR7. We found that MAVS^−/−^ and TLR7^−/−^ mice were both resistant to rJHMV_PS−_. These data indicate that neither TLR7- nor MAVS-mediated signaling was solely responsible for the attenuation of rJHMV_PS−_. Furthermore, MAVS^−/−^ mice treated with IFNAR-blocking antibody were more susceptible to rJHMV_PS−_ than control antibody-treated mice, suggesting that IFN-I was still responsible for rJHMV_PS−_ attenuation in these mice. Together, these data support a role for IFN-I signaling in rJHMV_PS−_ attenuation and suggest that TLR7 and MDA5 have a redundant function(s) or that other sensors/inflammatory pathways play a role in MHV packaging mutant attenuation.

The sgRNAs could act as PAMPs during the replication and/or entry of MHV packaging mutants. The presence of −sgRNAs in virions, albeit in small amounts, might be most important for the induction of IFN-I since negative-strand viral RNA is presumably not capped but, rather, contains a triphosphate at the 5′ end. This would facilitate recognition by the intracellular helical receptor RIG-I. Negative-sense sgRNA is expected to be present as double-stranded RNA (dsRNA), which could be detected by RIG-I, TLR3, and protein kinase R (PKR), in addition to MDA5. It should be noted that while our data suggest that IFN-I signaling and perhaps other changes in the innate immune response are largely responsible for rJHMV_PS−_ attenuation, defects in virus replication in cells in the infected brain may also contribute to attenuation. We observed no differences between the replication kinetics of rJHMV_PS−_ or rJHMV_Nsp15-HA_ and those of rJHMV_WT_ *in vitro* (Fig. S1), but this does not preclude subtle differences in primary cells, such as BMMs (Fig. 3D).

In conclusion, our results demonstrate that the selective packaging of gRNA plays an important role in the virulence of MHV and probably other lineage A betacoronaviruses. This work uncovers a unique method by which an RNA virus shields its RNA products from the host to prevent an innate immune response. Selective packaging is conserved among coronaviruses and therefore is likely important for the virulence of most, if not all, coronaviruses. However, the localization of the packaging signal for most coronaviruses is unknown. Thus, an important goal for future research will be to identify the packaging signals in pathogenic human and animal coronaviruses and determine their roles in virulence.

## MATERIALS AND METHODS

### Cell culture.

17Cl-1 cells, HeLa cells expressing MHV receptor carcinoembryonic antigen-related cell adhesion molecule 1 (CEACAM1a) (HeLa-MHVR), and baby hamster kidney cells expressing CEACAM1a (BHK-MHVR) were grown in Dulbecco’s modified Eagle’s medium (DMEM), with 10% fetal calf serum, 1% penicillin-streptomycin (Pen-Strep), 2% sodium bicarbonate, 2% l-glutamine, 1% nonessential amino acids, and 1% sodium pyruvate (D10). Low-serum medium is the same as D10 with 2% fetal calf serum (D2).

### Mice.

Pathogen-free C57BL/6 (B6) and BALB/c mice were purchased from Charles River Laboratories. B6 interferon alpha/beta receptor-knockout (IFNAR^−/−^) mice were obtained from the Jackson Laboratory, and Toll-like receptor 7-knockout (TLR7^−/−^) mice were obtained from Shizuo Akira (Osaka University) via Westley Reeves (University of Florida). Mice were bred in the animal care facility at the University of Iowa. Animal studies were approved by the University of Iowa Animal Care and Use Committee (IACUC).

### Generation of recombinant viruses.

pBAC-rJHMV_PS−_ and pBAC-rJHMV_Nsp15-HA_ were created using pBAC-rJHM.IA, by Red recombination, and using the primers listed in Table S1 in the supplemental material. Using gBlock fragments with the corresponding mutations and listed primers, a PCR product was created with the HA or PS^−^ substitution, kanamycin followed by an ISCE-I restriction site (KanI), and homologous arms. Either the KanI-HA- or KanI-PS^−^-containing product was transformed into competent GS1783 cells harboring the rJHMV.IA pBAC. Chloramphenicol-positive (CML^+^) Kan^+^ colonies were screened for intermolecular recombination by PCR and restriction enzyme digestion. Colonies with the correct intermediates were then used for intramolecular recombination of the homologous arms. Correct colonies were amplified, and the ISCE-I enzyme was induced with 1% arabinose for 2 h. The Lambda Red recombination enzymes were induced by heat shock at 42°C for 30 min, and cells were then incubated at room temperature for 4 h. CML^+^ Kan^−^ colonies were screened for correct final products by PCR, restriction enzyme digestion, and sequencing. pBAC.IA_PS−_ or pBAC.IA_Nsp15-HA_ was cotransfected with plasmid expressing N protein into BHK-MHVR cells using Lipofectamine 2000 (Fisher Scientific) according to the manufacturer’s protocol. Transfected BHK cells were collected at the time of maximal cytopathic effect and lysed by freeze-thawing. rJHMV_PS−_ and rJHMV_Nsp15-HA_ were then passaged 3 times to obtain working stocks. Two independent clones of the PS^−^ virus (Fig. 1C), rJHMV_PS−_, were isolated, and the sequence of each clone was confirmed. No coding mutations were present in either clone; however, rJHMV_PS−3.2_ did contain a synonymous mutation in nsp15 3′ of the PS. It is unlikely that this mutation significantly alters the secondary structure in this region, and this isolate behaved identically to rJHMV_PS−4_._2_. rJHMV_Nsp15-HA_ was sequenced after passaging with no mutations found.

10.1128/mBio.00272-18.4 TABLE S1  Primers used to engineer recombinant viruses. Download TABLE S1, DOCX file, 0.2 MB. Copyright © 2018 Athmer et al. 2018 Athmer et al. This content is distributed under the terms of the Creative Commons Attribution 4.0 International license.

To introduce the previously published PS^−^ mutation (8) into rA59, the published mutations were introduced into the F plasmid of the rA59 system (21). The F plasmid was linearized, and homologous arms, with the PS^−^ mutations, were added by PCR using the primers listed in Table S1. The linearized plasmid was recombined using the homologous arms and the In-Fusion cloning kit (Clontech) according to the manufacturer’s protocol. The PS^−^ mutations were screened for by PCR and sequencing. The F plasmid and other fragments were digested with BsmBI and ligated together using T4 DNA ligase. Ligated DNAs and N protein transcript were transcribed *in vitro* using the mMessage mMachine T7 transcription kit (Fisher Scientific) according to the manufacturer’s protocol. BHK-MHVR cells were then transfected by electroporation with transcribed RNAs. Transfected BHK-MHVR cells were plated in D10 and monitored for cytopathic effects. Rescued recombinant viruses were collected, their titers were determined as described above, and they were termed rA59_PS−_ P0. rA59_PS−_ P0 stocks were used to generate rA59_PS−_ P1 stocks by infecting 17Cl-1 cells with virus at a multiplicity of infection (MOI) of 0.1 PFU/cell and collecting cells at times of maximal cytopathic effects. Titers of P1 stocks of rA59_WT_ and rA59_PS−_ were determined, and the stocks were utilized for all experiments with rA59.

### Tissue culture cell infection.

Unless otherwise noted, all infections were completed as follows. Semiconfluent monolayers of cells were infected on either 12-well or 24-well plates. Virus was adsorbed in DMEM at 37°C for 30 min at the indicated MOI. After adsorption, remaining virus was removed, and the cells were returned to growth medium for the indicated times.

### Mouse infection.

For rJHMV infections, 5- to 8-week-old male mice were sedated using isoflurane and infected by the intranasal route with 3 × 10^4^ PFU, unless otherwise stated, in 12 µl. In IFNAR-blocking experiments, 1.5 µg of InVivoMAb (Bio X Cell; MAR1-5A3) IFNAR-blocking antibody or rat IgG (MP Biomedical) was administered by intraperitoneal (i.p.) injection 6 h prior to infection and 0.5 µg was administered 3 days postinfection. For rA59 infections, 5- to 8-week-old male mice were sedated and infected intracranially with 700 PFU. All infected mice were weighed and monitored for 2 weeks and euthanized if the mouse dropped below 70% of initial body weight or became dehydrated, in accordance with University of Iowa IACUC-approved animal protocols.

### RT-qPCR.

For cellular RNA experiments, RNA was isolated from cultured cells using Trizol (Fisher Scientific). cDNAs for all RNAs except sgRNAs (see below) were prepared using Moloney murine leukemia virus (MMLV) reverse transcriptase with random hexamers according to the manufacturer’s instructions. Real-time quantitative PCR (RT-qPCR) was performed using Applied Biosystems QuantStudio 3 and PowerUp Sybr green master mix (Applied Biosystems). All primers used for qPCR are listed in Table S2. Target gene levels were normalized to hypoxanthine-guanine phosphoribosyltransferase (HPRT) by the threshold cycle (*C*_*T*_) equation: Δ*C*_*T*_ = *C*_*T*_ of the gene of interest − *C*_*T*_ of HPRT. All results are shown as a ratio to HPRT calculated as 2^−Δ*CT*^.

10.1128/mBio.00272-18.5 TABLE S2  Primers used for qPCR. Download TABLE S2, DOCX file, 0.1 MB. Copyright © 2018 Athmer et al. 2018 Athmer et al. This content is distributed under the terms of the Creative Commons Attribution 4.0 International license.

### Selective viral RNA packaging.

Infected 17Cl-1 supernatants were collected at 20 hpi, when maximal cytopathic effects were observed but before cell lysis. Supernatants were clarified by centrifugation at 1,000 × *g* and filtered through a 0.45-µm filter. Clarified supernatants were overlaid on 30% sucrose in Na-Tris buffer (100 mM NaCl, 10 mM Tris-Cl, pH 8.0) and centrifuged at 27,000 rpm for 4 h at 4°C. Pelleted virions were resuspended in DMEM. Viral RNA and cellular RNA from infected monolayers were isolated using Trizol (Fisher Scientific) according to the manufacturer’s protocol. cDNA for viral RNAs was generated using Superscript IV (Fisher Scientific) and strand-specific primers (Table S2) according to the manufacturer’s protocol, while cDNA for HPRT was generated using random hexamers as described above. Quantification for viral RNAs was performed utilizing a standard curve, which was generated from reverse-transcribed PCR-derived gRNA and sgRNA products. Relative quantification was performed using Δ*C*_*T*_ with normalization to gRNA. Data are presented as a ratio of +/−sgRNA7 RNA to gRNA, gRNA units per milliliter, or HPRT RNA to gRNA.

### Viral titers.

Viral titers were determined by infecting HeLa-MHVR cells as described above. Following rA59 infection, a 1:1 ratio of 1.2% agarose and D2 was applied to cells and the cells were incubated for 24 h at 37°C. Following incubation, cell monolayers were fixed with 4% formaldehyde in phosphate-buffered saline (PBS) and stained with 0.1% crystal violet in 2% methanol. Following rJHMV infection, cells were returned to D2 medium for 16 h at 37°C. After incubation, a 1:1 mixture of 1.2% agarose and D2 with 1% neutral red was applied for 4 h at 37°C before counting plaques.

### BMM cultures.

Male and female B6 mice were euthanized, and bone marrow was isolated from each femur and tibia. The bone marrow was treated with ammonium-chloride-potassium lysing buffer for 30 s and then washed three times with RPMI containing 10% fetal calf serum. Bone marrow cells were then strained through 70-µm strainers and counted. Bone marrow cells (5 × 10^5^) were plated in BMM medium (RPMI, 10% fetal calf serum, 10% L929 cell supernatant, 1% Pen-Strep, 1% sodium pyruvate, 1% l-glutamine) and cultured for 4 days. Following the 4th day, medium was changed daily until the day of experiment (day 7). BMMs were infected as described above at an MOI of 1 PFU/cell.

### Statistics.

Student’s unpaired *t* test or the Mann-Whitney U test was used to analyze differences in mean values between groups. All results are expressed as mean ± range or ±standard error of the mean where indicated. *P* values of ≤0.05 were considered statistically significant. Survival data were analyzed using Mantel-Cox tests (*, *P* < 0.05).
