# Supplementary figures and images for: Selective Packaging in Murine Coronavirus Promotes Virulence by Limiting Type I Interferon Responses
Source: mBio. 2018 May 1;9(3):e00272-18. doi: 10.1128/mBio.00272-18 (PMC5930304; doi:10.1128/mBio.00272-18)

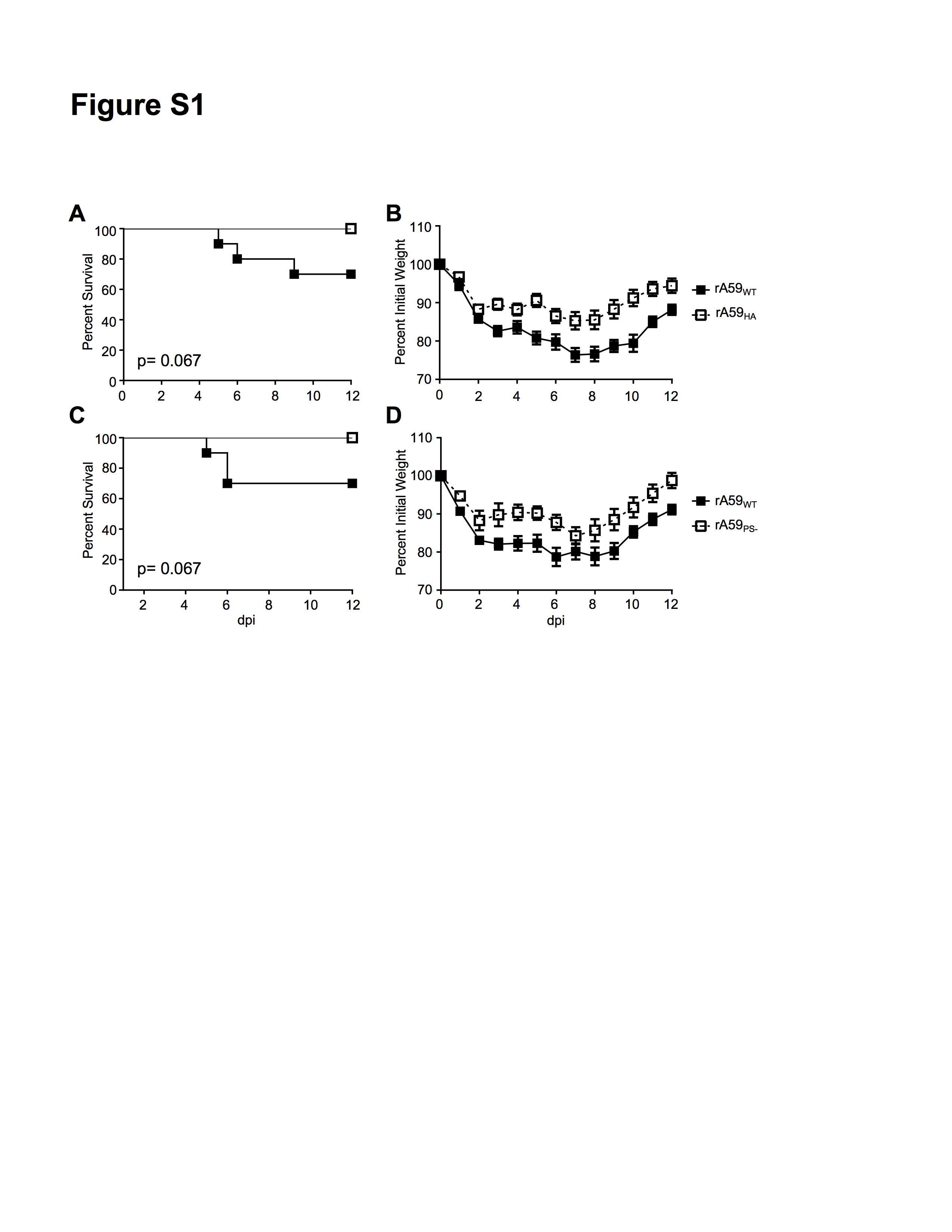

Supplement: FIG S1 [file mbo002183864sf1.tif]

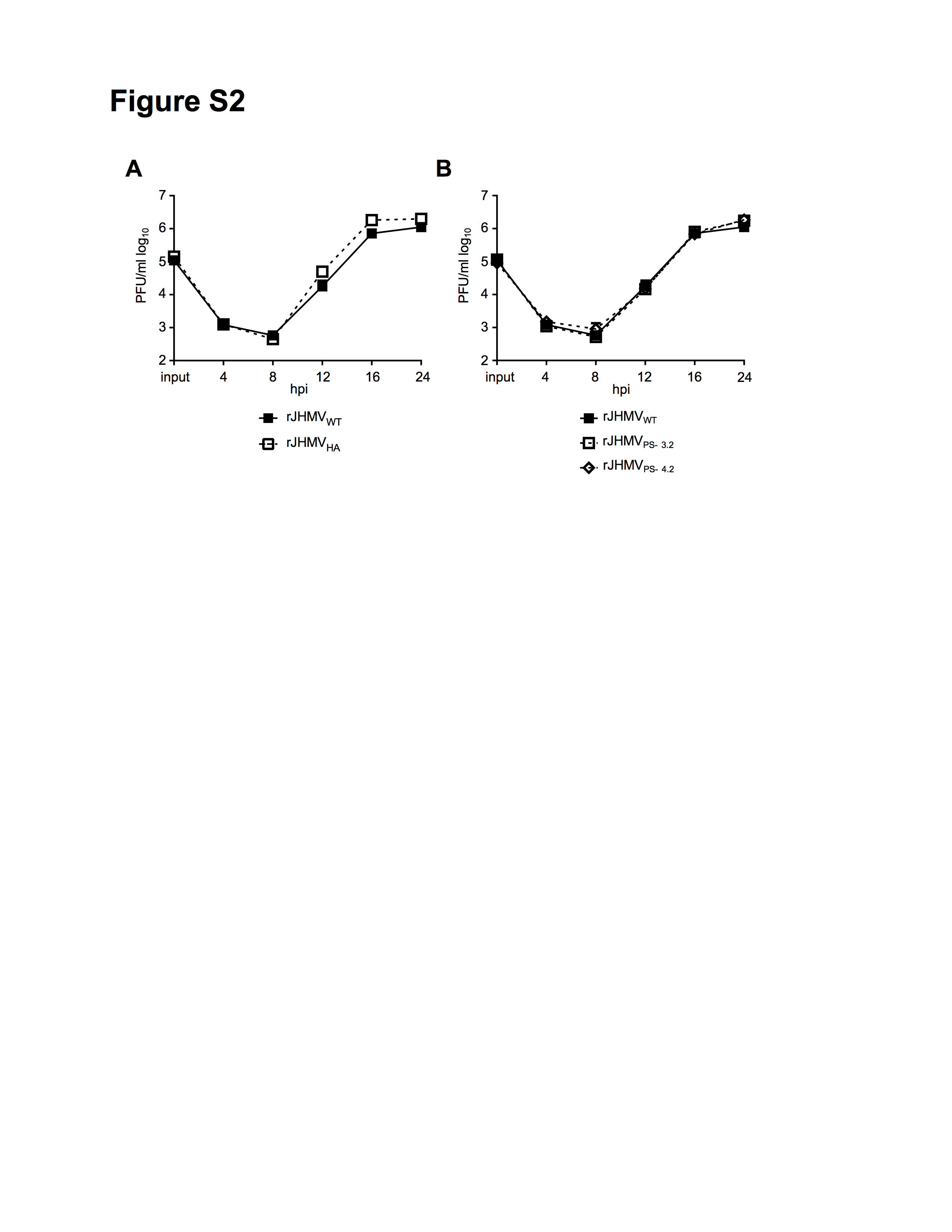

Supplement: FIG S2 [file mbo002183864sf2.tif]

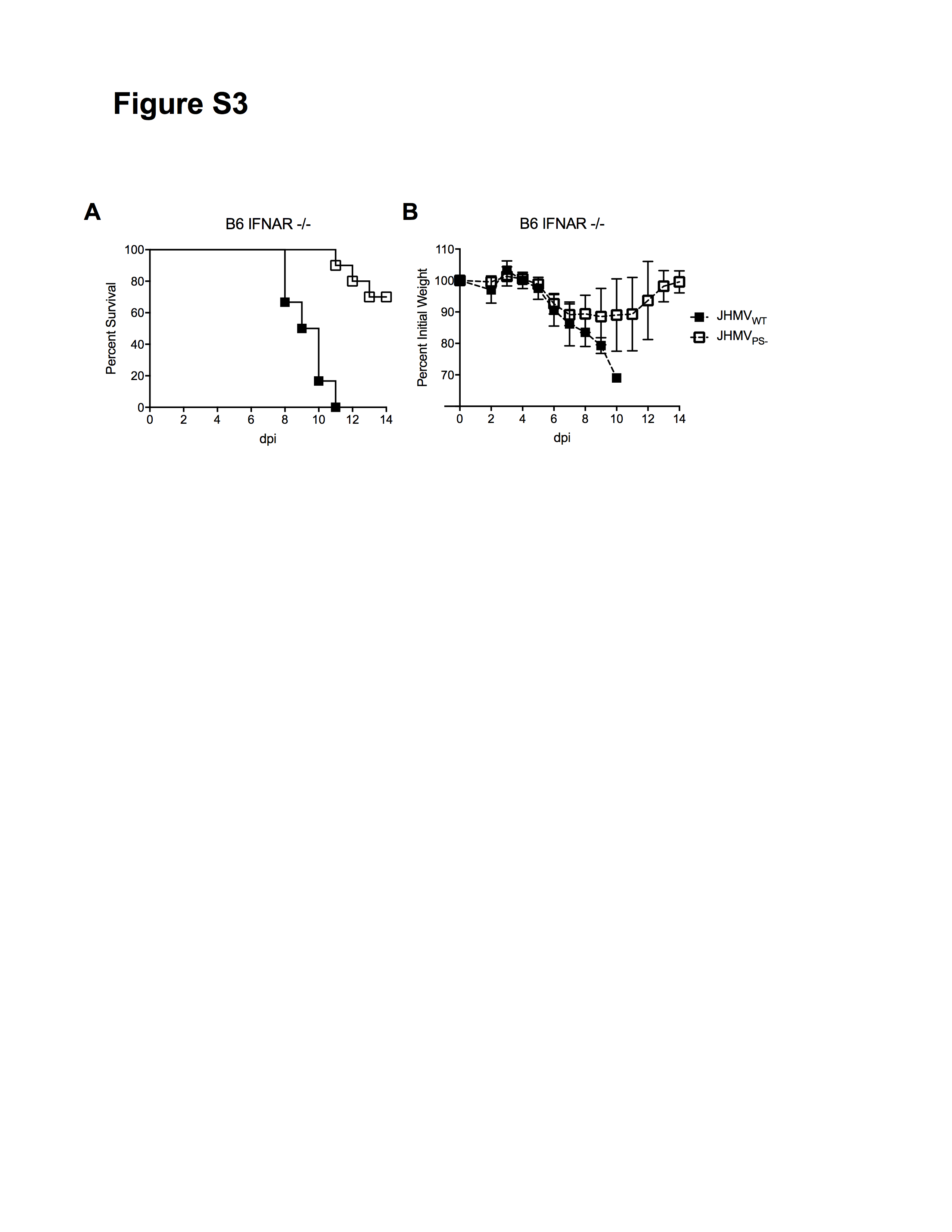

Supplement: FIG S3 [file mbo002183864sf3.tif]

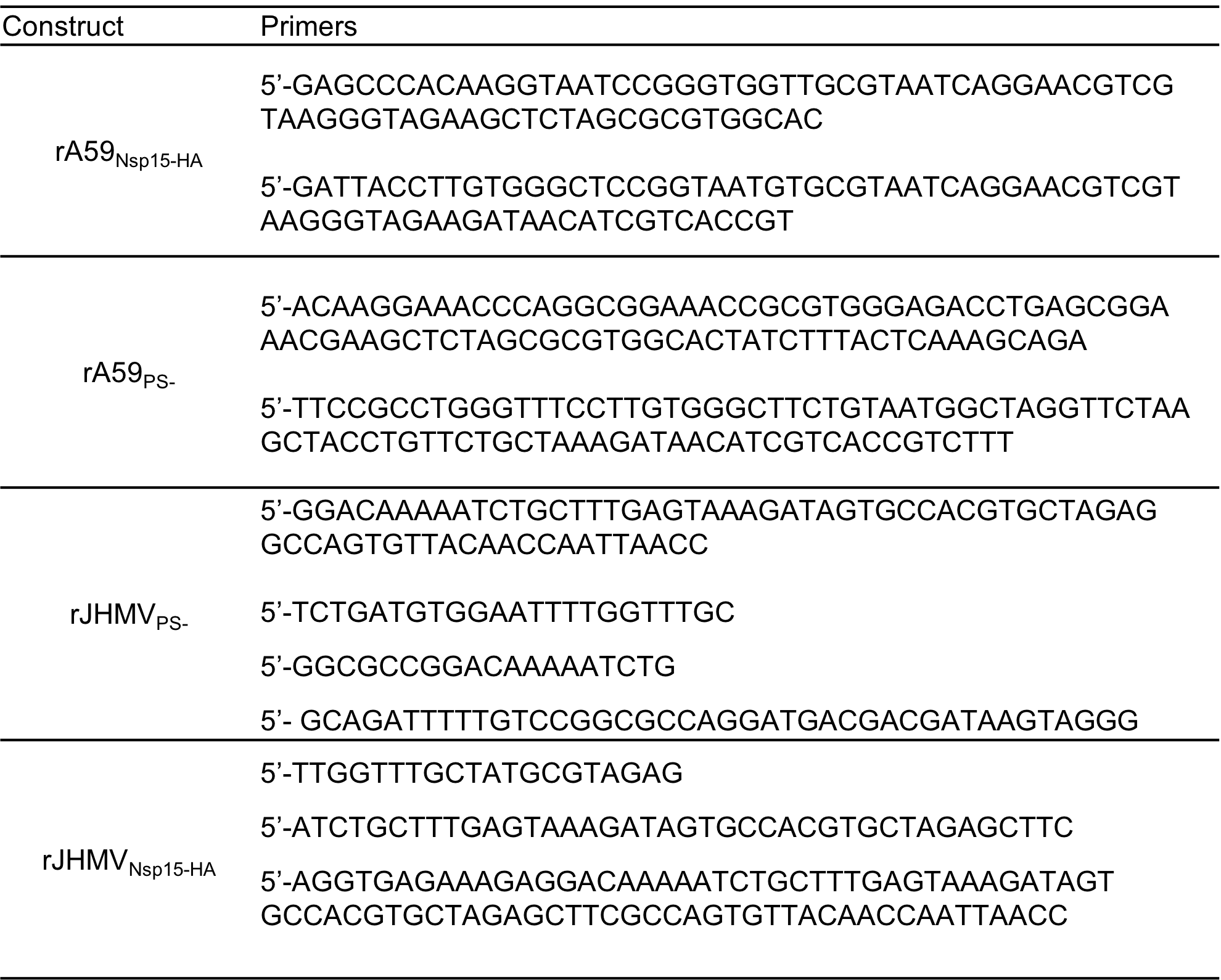


Table S1: Primers used to engineer recombinant viruses

Supplement: TABLE S1 [file mbo002183864st1.docx]

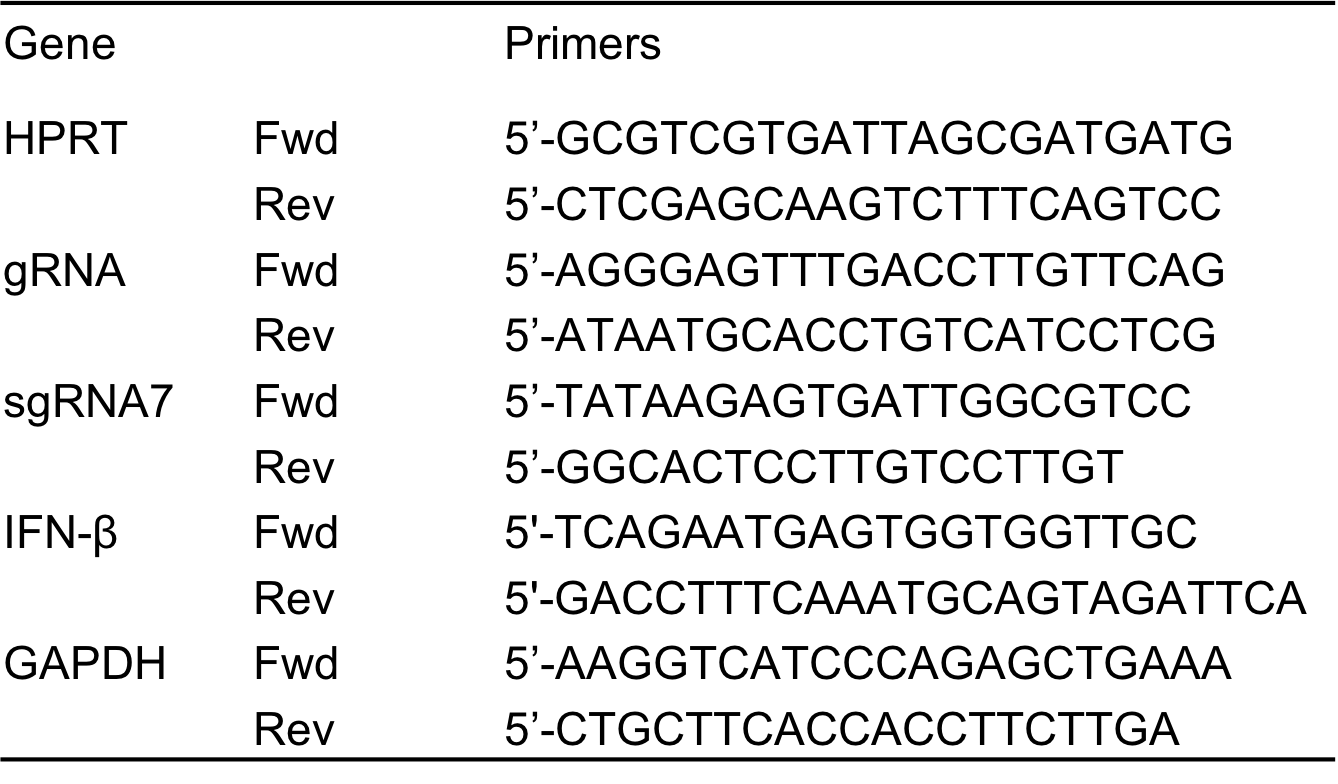


Table S2: Primers used for qPCR

Supplement: TABLE S2 [file mbo002183864st2.docx]
